# Comparative Study of Acid Etching and SLA Surface Modification for Titanium Implants

**DOI:** 10.3390/ma18071632

**Published:** 2025-04-03

**Authors:** Gabriel M. Vieira, Tatiane C. S. Almeida, Fernanda P. Oliveira, Patrícia C. Azzi, Caio F. Rodrigues, Rafael L. Souza, Samyra Maria S. N. Lacerda, Frederico S. Lages, Maximiliano D. Martins

**Affiliations:** 1 Centro de Desenvolvimento da Tecnologia Nuclear (CDTN), Belo Horizonte 31270-901, MG, Brazil; tatyanealmeyda@gmail.com (T.C.S.A.); nandadepaula116@hotmail.com (F.P.O.); patriciacanazart@gmail.com (P.C.A.); fabrinircaio@gmail.com (C.F.R.); 2 Instituto de Engenharia, Ciência e Tecnologia, Universidade Federal dos Vales do Jequitinhonha e Mucuri (UFVJM), Janaúba 39440-000, MG, Brazil; rafael.souza@ufvjm.edu.br; 3 Departamento de Odontologia Restauradora, Universidade Federal de Minas Gerais (UFMG), Belo Horizonte 31270-901, MG, Brazil; samyranassif@gmail.com; 4 Departamento de Morfologia do Instituto de Ciências Biológicas, Universidade Federal de Minas Gerais (UFMG), Belo Horizonte 31270-901, MG, Brazil; fredlages@hotmail.com

**Keywords:** dental implants, surface-active agents, titanium, cell adhesion, osseointegration

## Abstract

The dust generated during the sandblasting process of the sandblasted and acid-etched (SLA) method, commonly used to treat the surface of Ti dental implants, poses significant challenges in maintaining a clean manufacturing environment and ensuring safe working conditions. Nevertheless, surface modification remains crucial for improved performance of Ti dental implants. To address this problem and propose a clean and simple surface modification process to potentially replace SLA modification, this study aimed to characterize the surfaces of commercially pure Ti (cp-Ti) samples treated by acid etching and compare them with SLA-treated samples in terms of surface roughness (R_q_), wettability (assessed through contact angle measurements), mineralized matrix deposition (evaluated through simulated body fluid [SBF] soaking), cell viability, cell differentiation (assessed based on alkaline phosphatase activity), and mineralization (assessed using MTT assay). Acid-etched surfaces exhibited nano- and micro-roughness and higher hydrophilicity than SLA surfaces, which is conducive to forming a highly bioactive TiO_2_ surface. Moreover, acid-etched samples exhibited earlier hydroxyapatite deposition after SBF soaking than SLA samples. Furthermore, the acid-etched surfaces were nontoxic and displayed significantly higher cell viability and differentiation after seven days than SLA surfaces. These findings suggest that acid etching is a viable alternative to the SLA method, likely offering superior surface bioactivity and biocompatibility.

## 1. Introduction

The global average life expectancy is increasing annually. As the population ages, age-related health issues, including tooth loss due to periodontal disease, dental caries, and loss of bone-recovery capacity, have become more prevalent, leading to a growing worldwide demand for dental implants [1,2]. Since the 1980s, commercially pure titanium (cp-Ti) and titanium alloys such as Ti-6Al-4V have been established in the market as the primary materials for dental implants [3,4]. The biocompatibility of Ti and its alloys is linked to the spontaneous formation of a passivating titanium dioxide (TiO_2_) layer on their surfaces [5]. This layer imparts chemical properties such as inertness and high corrosion resistance in air and biological fluids [6]. Additionally, Ti possesses mechanical properties that promote its use in biological implants, including a favorable weight-to-strength ratio, a modulus of elasticity relatively close to that of human bone, and excellent workability, that is, ease of mechanical processing [7,8,9].

A recurring problem experienced by patients after surgery for the insertion of metallic dental implants is the intermediate formation of soft fibrous tissue between bone and the implant, which delays or prevents implant anchorage in the bone matrix [8,10]. Softening or inflammation around the implant results from poor osseointegration, leading to implantation failure. Thus, assessing implantation success requires ensuring that the implant remains securely anchored, stable, and biologically inert [11,12,13].

Over the past decades, various surface modification methods have been developed to enhance osseointegration in dental implants. Among these methods, physicochemical treatments, such as anodization, allow the formation of nanotubular TiO_2_ layers, increasing the surface area. Mechanical treatments, such as sandblasting, introduce micro-roughness by bombarding the surface with abrasive particles. The sandblasted and acid-etched (SLA) method is a combination of mechanical and chemical surface modifications and is used worldwide. However, the use of SLA is challenged by practical inconveniences related to contamination levels in the production site due to the use of blasting microparticles, and acid etching alone is a simpler chemical treatment capable of generating micro- and nano-scale roughness, significantly enhancing cell adhesion, corrosion resistance, and osseointegration [13,14,15].

The presence of micro- and nano-textures, also known as micro- and nano-roughness, on the surface of a dental implant plays a crucial role in protein-adsorption, which initiates cell adhesion and mediates the biological events responsible for osseointegration [15,16,17]. In addition, surfaces with lower contact angles and greater wettability increase body-fluid contact with the implant surface, facilitating osseointegration [18].

Acid etching involves immersing implants in one or more acidic solutions, such as sulfuric, nitric, or hydrofluoric acids [16]. This process creates micro-sized pits, thereby generating rougher surfaces than machined surfaces. This treatment accelerates bone ingrowth and enhances biological adhesion, which improves the long-term success rate of implants [16,17]. Furthermore, acid-etching methods are inherently easier to implement than the SLA method, which requires grit blasting followed by acid etching. In addition, acid-etching treatments have a lower risk of implant contamination, as no blasting particles remain on the surface [17]. This study aimed to characterize the surfaces of cp-Ti samples subjected to different surface modifications via acid etching and to compare the results with those of SLA-treated samples. The null hypothesis was that no significant changes in surface morphology, roughness, or in vitro assays such as cell viability, cell differentiation, and mineralized matrix would be observed when comparing cp-Ti surfaces modified via SLA and acid etching.

## 2. Materials and Methods

### 2.1. Sample Preparation and Surface Treatments

**Figure 1:**
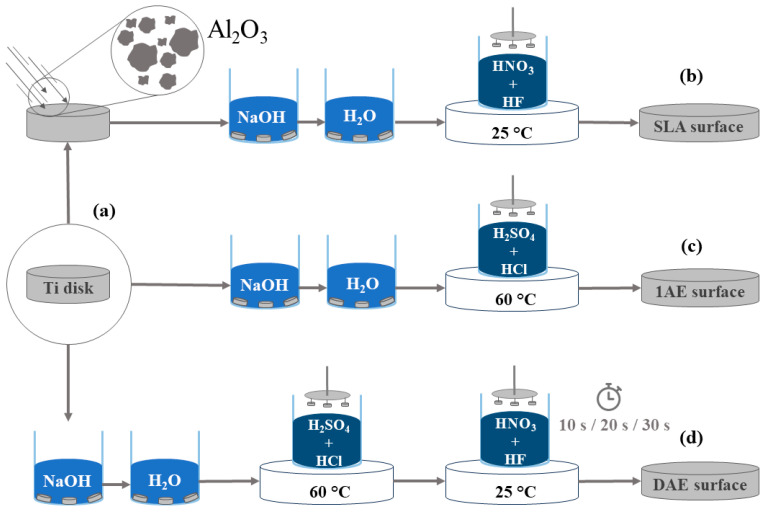
Schematic of the experimental work carried out in this study. (**a**) Ti samples; (**b**) SLA surface; (**c**) surface obtained after first acid etching; (**d**) surfaces obtained after second acid etching for 10, 20, and 30 s. SLA, sandblasted and acid-etched; 1AE, single-acid-etched; DAE, double-acid-etched.

Figure 1 presents a schematic of the experimental work. Samples of the cp-Ti ASTM F67 [19] grade 4 were machined into disks of 9.5 mm diameter and 5 mm thickness. Surface-modification protocols were performed at PecLab Ltda. to generate SLA and acid-etched samples (Belo Horizonte, MG, Brazil). Specific information, including the concentrations of the acidic solutions, is not presented owing to proprietary industrial confidentiality. The samples were separated into five distinct groups. The SLA-treated samples; 1AE group, submitted to H_2_SO_4_ and HCl acid etching; and the groups DAE-10, DAE-20, and DAE-30, submitted to H_2_SO_4_ and HCl etching, followed by HNO_3_ and HF etching, as listed in Table 1.

### 2.2. Roughness and Hydrophilicity Assessment

The roughness parameter R_q_ of the TiO_2_ surfaces was determined from the linear roughness profiles, obtained using atomic force microscopy (AFM; NTREGRA, NT-MDT, Moscow, Russia), and for each sample group, we used Gwyddion software (2.67 version) [20]. The contact angles were measured using the sessile drop method (with a drop volume of 3 µL), at 25 ± 2 °C in ambient atmosphere. The samples were previously placed in an oven for 90 min at 160 ± 2 °C to mitigate water surface adsorption. The measured angle was formed between the horizontal (base of the water drop) and tangent lines of the drop surface.

### 2.3. Bioactivity Assessment by Immersion in Simulated Body Fluid

Hydroxyapatite (HA) deposition was performed by sample immersion in Kokubo’s simulated body fluid (SBF), a supersaturated solution of calcium, carbonate, and phosphate ions, first proposed by Kokubo et al. [21]. The soaking times were 3, 7, 14, 21, and 28 days, using a shaker, model Innova (New Brunswick Scientific, Edison, NJ, USA), at 36.5 °C and 60 rpm. HA deposition was evaluated using scanning electron microscopy (SEM), energy-dispersive X-ray spectroscopy (EDS; MEV/FEG Quanta 200, FEI, Hillsboro, OR, USA), and X-ray diffraction analysis (XRD; Ultima IV; Rigaku, Tokyo, Japan).

### 2.4. In Vitro Study

To reduce the number of samples for the in vitro assays, the double-acid-etching (DAE) group that performed best in the bioactivity assessment was selected for comparison with the SLA and single-acid-etching (1AE) groups.

### 2.5. Cell Culture

Cells from the MC3T3-E1 subclone 14 line, immortalized pre-osteoblasts from the skull of neonate mice (American Type Culture Collection, Arlington, VA, USA), were cultured in Alpha Minimum Essential Medium (α-MEM; Thermo Fisher Scientific, Waltham, MA, USA) supplemented with 10% fetal bovine serum (FBS) (Gibco^TM^, Thermo Fisher Scientific Waltham, MA, USA), antibiotics streptomycin (100 μg/mL), and penicillin (500 U/mL) (Invitrogen^TM^, Thermo Fisher Scientific, Waltham, MA, USA). The culture medium was changed every 48 h until the cells reached confluence. The cells were collected using a solution of trypsin (0.25%) and ethylenediaminetetraacetic acid (Gibco^TM^, Thermo Fisher Scientific, Waltham, MA, USA). Subsequently, the cells were resuspended in fresh medium and transferred to new flasks at the desired ratio (e.g., 1:5). The experiments were performed using the 20th passage, as osteoblasts in continuous passages show reduced mineralized matrix formation [22].

To perform in vitro assays, three samples from each group were placed in a 48-well plate, and MC3T3-E1 pre-osteoblast cells were seeded at 1 × 10^4^ density per well. After plating, they were incubated in an oven at 37 °C in a humid atmosphere containing 5% CO_2_. For the control group, all the procedures in each in vitro assay were performed in empty wells. The samples were sterilized by gamma irradiation at a dose of 12 kGy using a cobalt-60 gamma source (model IR-214, type GB-127, Nordion Inc., Ottawa, ON, Canada) at the Gamma Irradiation Laboratory of the Centro de Desenvolvimento da Tecnologia Nuclear.

### 2.6. Cell Viability Assay

The cytotoxic effects of the samples were evaluated using a 3-(4,5-dimethylthiazol-2-yl)-2,5-diphenyl-2H-tetrazolium bromide (MTT) assay (Life Technologies, Carlsbad, CA, USA). The assessments were performed over 24 and 48 h [23].

After removing the treatment medium, basal α-MEM was added along with 100 μL of MTT (5 mg/mL) per well, totaling 130 μL. Following a 2 h incubation at 37 °C in a humidified atmosphere with 5% CO_2_, formazan crystals were observed and dissolved in 130 μL of 10% sodium dodecyl sulfate (SDS) in HCl 0.01 M (Sigma–Aldrich^®^, Saint Louis, MO, USA). All steps were performed on culture plates.

After 18 h, the well contents were transferred to labeled centrifuge tubes and centrifuged at 2000 rpm for 2 min. Next, 100 μL of the centrifuged solution was placed in 96-well plates, and the optical density was measured at a wavelength of 595 nm using a spectrophotometer (uQuant, Biotek, Winooski, VT, USA). The mean cell viability of the experimental groups was normalized to that of the control group. All experiments were conducted in triplicate.

### 2.7. Cell Differentiation

MC3T3-E1 cells were induced to osteogenic differentiation by culturing them in supplemented α-MEM medium containing an osteogenic solution containing 2.165 mg/mL β-glycerolphosphate and ascorbic acid (Sigma–Aldrich^®^, Saint Louis, MO, USA). The culture plates were placed in a 5% CO_2_ incubator. The osteogenic differentiation medium was refreshed every two days.

### 2.8. Alkaline Phosphatase Activity

To investigate the contribution of the modified Ti surfaces in stimulating the differentiation of pre-osteoblastic cells, ALP activity, an early-stage marker of osteogenesis, was evaluated after days 3 and 7, with 7 days being the standard time reported in the literature for this differentiation to occur [24]. Alkaline phosphatase (ALP) activity was evaluated using the 5-bromo-4-chloro-3-indolyl phosphate (BCIP)-nitroblue tetrazolium salt (NBT) kit assay after 7 and 14 days of differentiation. The culture medium was discarded during the analysis period, and the samples were washed with PBS. After discarding the PBS, the cells were incubated with 200 µL/well of NBT/BCIP solution at a proportion of 1:1:8 in PBS for 2 h at 37 °C and 5% CO_2_. After confirming the presence of blue precipitates under an optical microscope, 210 µL/well of SDS was added, which smoothed the cells, and 10% HCl was added without removing the incubated NBT/BCIP solution. The plates were incubated overnight for 18 h to promote the solubilization of the precipitates. After this period, 100 µL of each well was transferred in triplicate to a 96-well plate, and the optical density was measured using a spectrophotometer (uQuant, Biotek, Winooski, VT, USA) at a wavelength of 595 nm.

### 2.9. Mineralization Assay

Mineralized matrix deposition was assessed using Alizarin red staining after 14 and 21 days of differentiation. Following this, the supernatant was collected, and the cells were rinsed with sterile 1× PBS (pH 7.4). Subsequently, the cells were fixed in a refrigerator using 70% ethanol for 1 h. The wells were then washed with distilled water, and a solution of Alizarin red dye (40 mmol/L, pH 4.2; Sigma–Aldrich^®^, St. Louis, MO, USA) was added (at a concentration of 40 mmol/L, pH 4.2) and stirred for 20 min. The dye was then removed, and the wells were washed again with distilled water.

For further processing, 200 µL of 10% cetylpyridinium chloride was added to each well and stirred for 30 min. After shaking, 100 µL from each well was transferred in triplicate to a new flat-bottom culture plate, and the spectrophotometer (uQuant, Biotek, Winooski, VT, USA) was used to measure the optical density at a wavelength of 550 nm. The entire assay was conducted in triplicate, and the results are expressed as the percentage of mineralized area relative to the total analyzed area.

### 2.10. Statistical Analysis

Statistical analyses were performed using GraphPad Prism software (version 8.0; GraphPad Software Inc., San Diego, CA, USA). Normality tests were performed to assess distribution. The data are expressed as mean ± standard error of the mean (S.E.M.). The data on both roughness and contact angle are represented as mean ± standard deviation and compared statistically using one-way analysis of variance (ANOVA) followed by Tukey’s post hoc test, with significance set at *p* < 0.05. The same statistical approach was applied to cell viability, alkaline phosphatase activity, and mineralization assay data, with a significance level of 5%.

## 3. Results

### 3.1. Roughness Assessment

**Figure 2:**
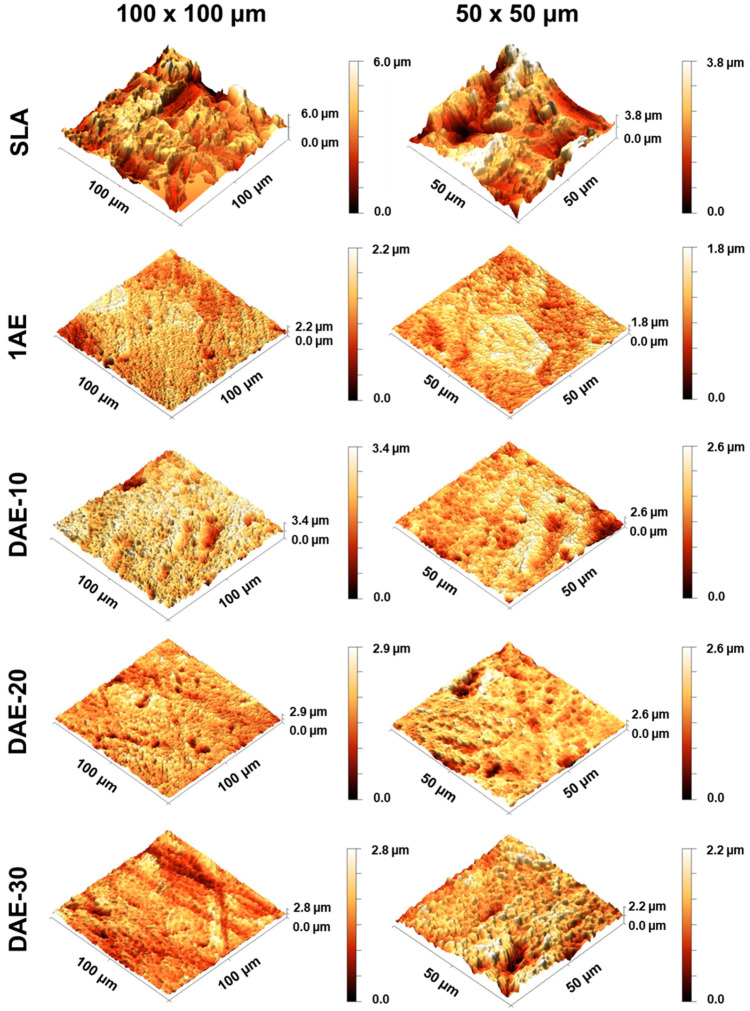
Atomic force microscopy three-dimensional semi-contact-mode topographic images of all sample groups. (**Left**) 100 µm^2^ images and (**right**) 50 µm^2^ images.

Figure 2 shows the AFM images of the investigated samples, which allow for a qualitative analysis of the topographic characteristics of each sample group. The SLA surface was significantly rougher than the machined and acid-etched surfaces. Additionally, surface roughness increased in DAE (H_2_SO_4_/HCl + HNO_3_/HF) samples compared to those in 1AE samples (H_2_SO_4_/HCl).

**Figure 3:**
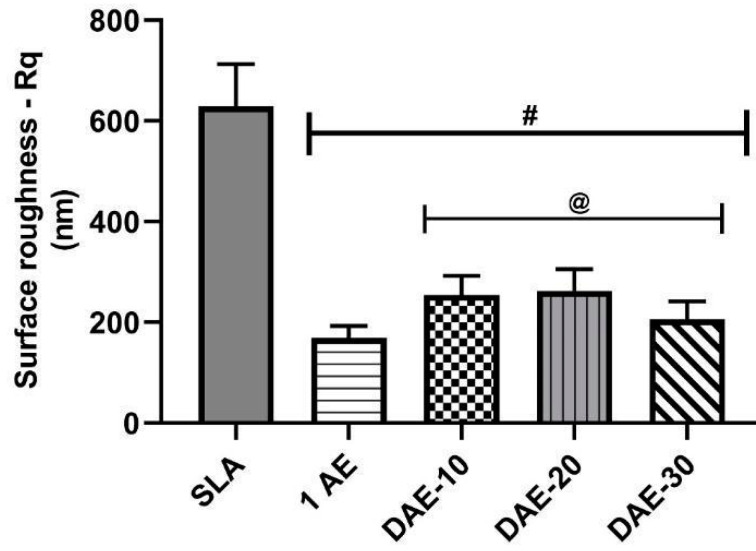
Roughness parameter R_q_ for all sample groups. # *p* < 0.05 vs. SLA; @ *p* < 0.05 vs. 1AE.

The roughness data in Figure 3 are presented in terms of the mean value and standard deviation. The R_q_ values were as follows: SLA group, 619 ± 95 nm; 1AE group, 169 ± 24 nm; DAE-10 group, 255 ± 38 nm; DAE-20 group, 263 ± 43 nm; and DAE-30 group, 206 ± 36 nm. ANOVA was performed for the five independent treatment groups with 120 roughness measurements each. Tukey’s test revealed a significant difference between the SLA group (#) and acid-etched groups, as well as between the 1AE (@) and DAE samples. DAE-10, DAE-20, and DAE-30 showed no significant differences in the roughness parameter R_q_.

**Figure 4:**
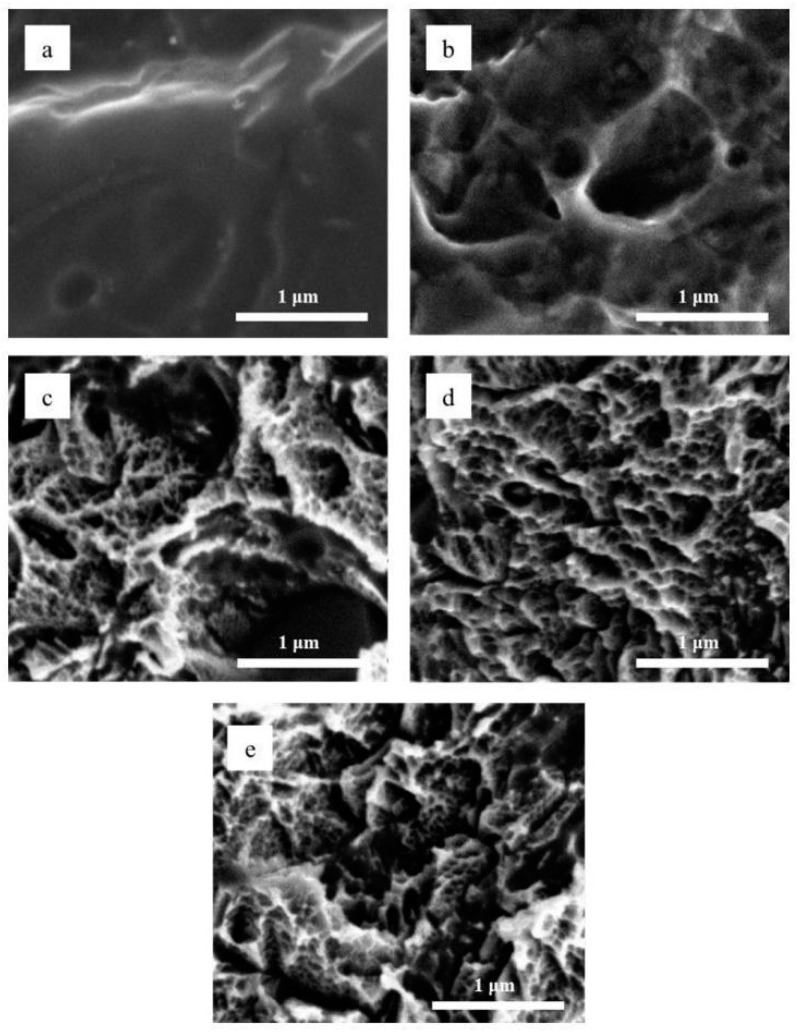
Scanning electron microscope images of the surfaces of the (**a**) SLA, (**b**) 1AE, (**c**) DAE-10, (**d**) DAE-20, and (**e**) DAE-30 samples.

**Figure 5:**
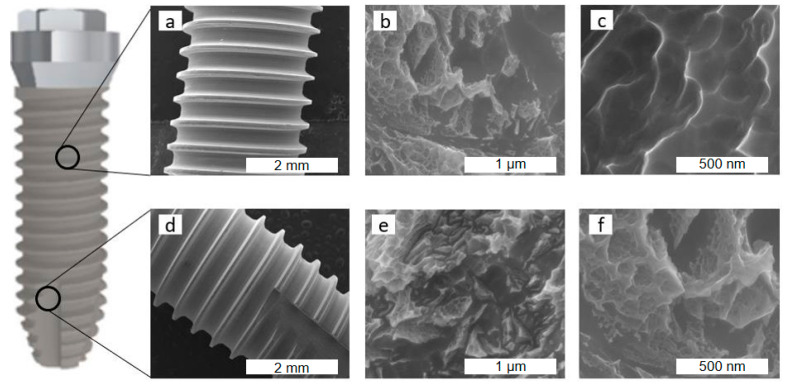
SEM images of different regions of a Ti dental implant treated with the same protocol as that of DAE-30. (**a**) Upper region of the implant-screw thread with a 30× magnification, (**b**) 50,000× magnification, and (**c**) 100,000× magnification and (**d**) lower region of the implant-screw thread with 30× magnification, (**e**) 50,000× magnification, and (**f**) 100,000× magnification. SEM—scanning electron microscope.

Figure 4 shows SEM images of the surface of all sample groups, and it is possible to notice that the SLA surface (Figure 5a) did not present sub-micron features as the acid-etched samples presented. The acid-etching protocol, the same as that used for DAE-30 group, was applied to a dental implant. The SEM images in Figure 5 demonstrate the reproducibility of the surface-modification process via the DAE of the dental implant. The implant used was an external hexagon connection, 4 × 12 mm (ID Maxi, PecLab Ltda, Belo Horizonte, MG, Brazil). This prosthetic connection was selected, as it is the most popular in the field of implantology and for being present in the catalog of most companies in the world. Two different regions of the implant were analyzed (the upper and lower regions of the implant screw thread), and micro- and nano-sized features were observed on the surface of both regions, similar to those in the acid-etched sample groups.

### 3.2. Surface Chemical Composition

**Figure 6:**
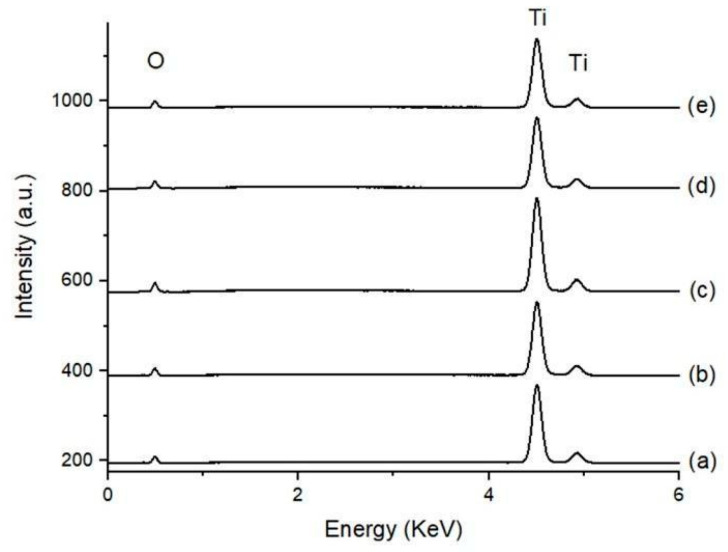
EDS spectra of all sample groups at a 15 keV Cu Kα energy beam; (a) SLA, (b) 1AE, (c) DAE-10, (d) DAE-20, and (e) DAE-30 samples. EDS—energy-dispersive X-ray spectroscopy.

Figure 6 shows the EDS spectra of the five sample groups. The signals in the energy ranges of Kα (4.508 keV) and Kβ (4.931) of Ti were predominant, and less intense signals corresponding to Lα of Ti and Kα of oxygen (0.452 keV and 0.525 keV, respectively) were identified.

### 3.3. Surface Wettability

**Figure 7:**
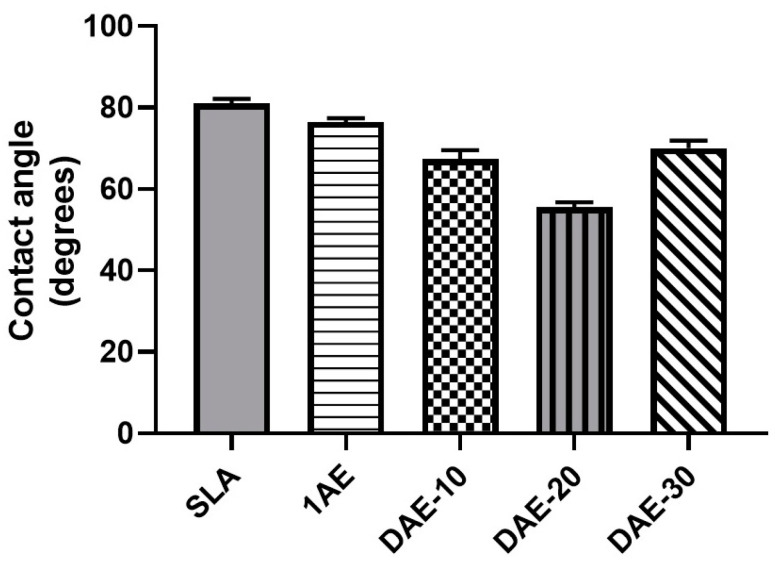
Contact angles based on the sessile water drop method for all sample groups.

All sample surfaces treated with DAE showed contact angles that were significantly lower (*p* < 0.05) than those of the SLA group. SLA group showed a contact angle of 80.9° ± 1.1°, whereas the acid-etched groups displayed lower contact angles of 76.4° ± 1.0°, 67.3° ± 2.3°, 55.5° ± 1.2°, and 70.0° ± 1.8°. The DAE-20 group exhibited the lowest contact angle (55.5° ± 1.2°). All groups showed statistically significant (*p* < 0.05) differences among each other, as shown in Figure 7.

### 3.4. Bioactivity Assessment by Immersion in SBF

**Figure 8:**
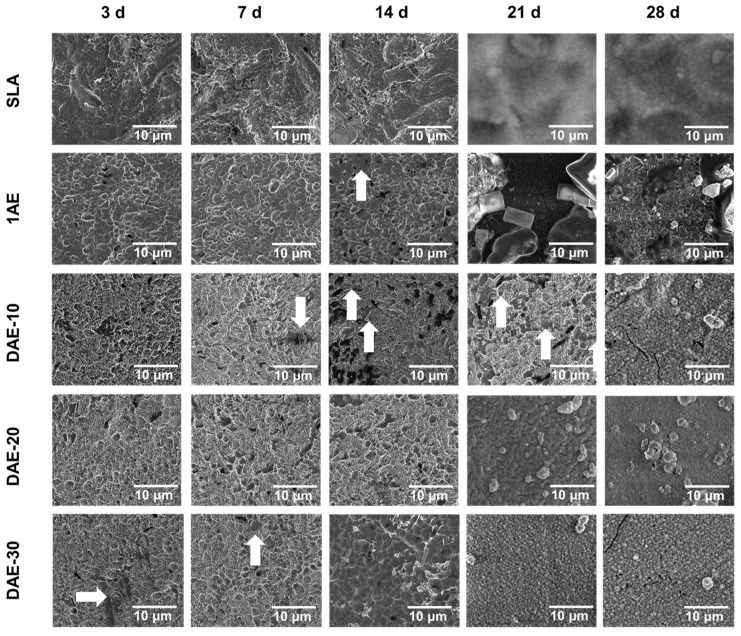
SEM images of all sample groups after 3, 7, 14, 21, and 28 days of SBF soaking. White arrows indicate spots that suggest an initial formation of a hydroxyapatite layer. SBF—simulated body fluid.

Immersion tests of the samples in SBF were conducted for the five sample groups at five different time intervals (3, 7, 14, 21, and 28 days) at 37 °C with continuous agitation at 60 rpm. An analysis of the SEM images (Figure 8) showed that the formation of mineral-deposition spots (indicated by the arrows) was observed, followed by a mineral-deposition layer, which was distinguished from the sample surface by different contrasts. As the immersion time increased, a gradual increase in the deposition coverage for all sample groups was observed. This ultimately led to complete surface coverage, which occurred for all groups at the 28-day mark.

Samples from the DAE groups (10 and 30 s) exhibited deposition spots by the 7th day. Moreover, the DAE-30 group was the only group in which mineral deposition began after the third day of soaking, and its surface was completely covered by spots after 14 days of soaking.

**Figure 9:**
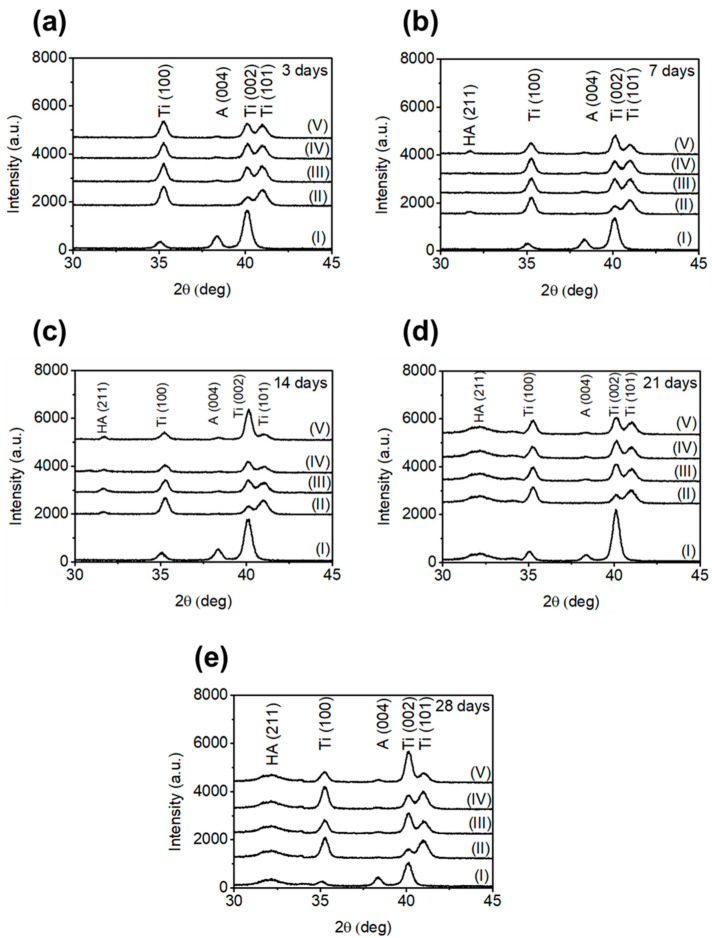
X-ray diffractograms of all sample groups after different periods of soaking. (**a**) 3 days, (**b**) 7 days, (**c**) 14 days, (**d**) 21 days, and (**e**) 28 days. (I) SLA, (II) 1AE, (III) DAE-10, (IV) DAE-20, and (V) DAE-30.

XRD was used to identify the composition of the deposited layers, represented qualitatively in Figure 9. The X-ray diffractograms revealed typical peaks of HA, metallic titanium (Ti), and anatase (A), as seen in Figure 8.

XRD results show the temporal evolution of the intensity of HA peaks in different sample groups. Consistent with the electron micrographs, the DAE-30 and 1AE samples showed the presence of HA as early as day 7. On the 14th day, all groups subjected to DAE (10, 20, and 30 s) exhibited a HA peak. In contrast, the SLA samples started displaying HA peaks only from the 21st day of immersion.

**Figure 10:**
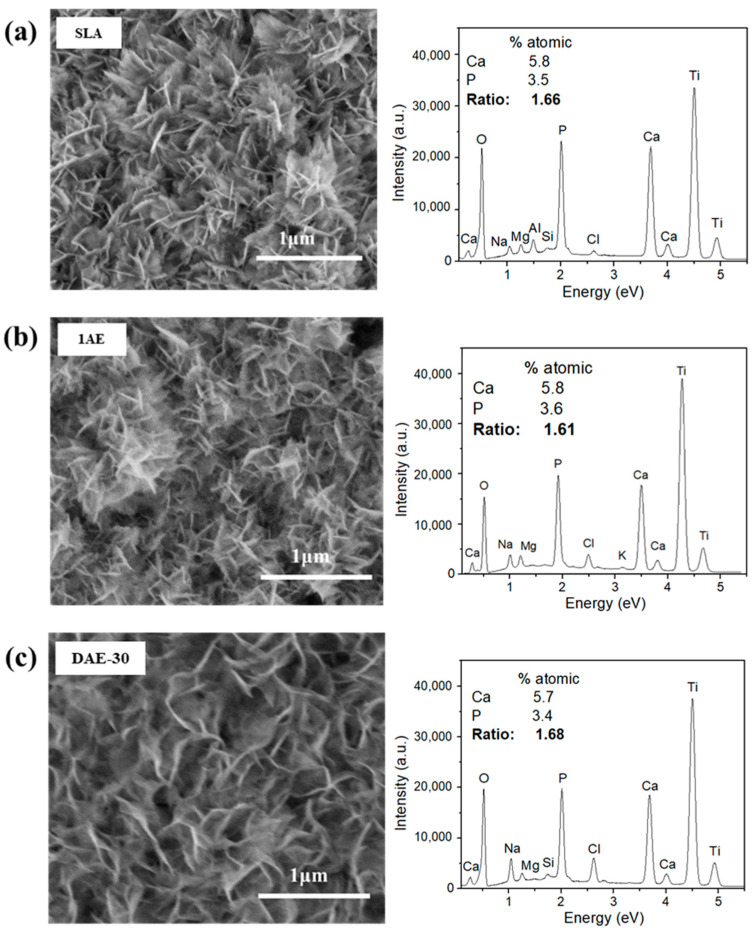
Images on the left show SEM images of sample surfaces after 28 days of SBF soaking, and images on the right show EDS spectra and the semi-quantitative analysis of atomic percentage for the (**a**) SLA, (**b**) 1AE, and (**c**) DAE-30 surfaces.

In addition to the electron micrographs and X-ray diffractograms, semi-quantitative EDS measurements were performed to verify the presence of HA by determining the atomic and mass percentages of calcium and phosphorus ions in the SLA, 1AE, and DAE-30 samples after 28 days of immersion in SBF. As shown in Figure 10, titanium, oxygen, calcium, and phosphorus were observed on the surface.

### 3.5. In Vitro Tests

**Figure 11:**
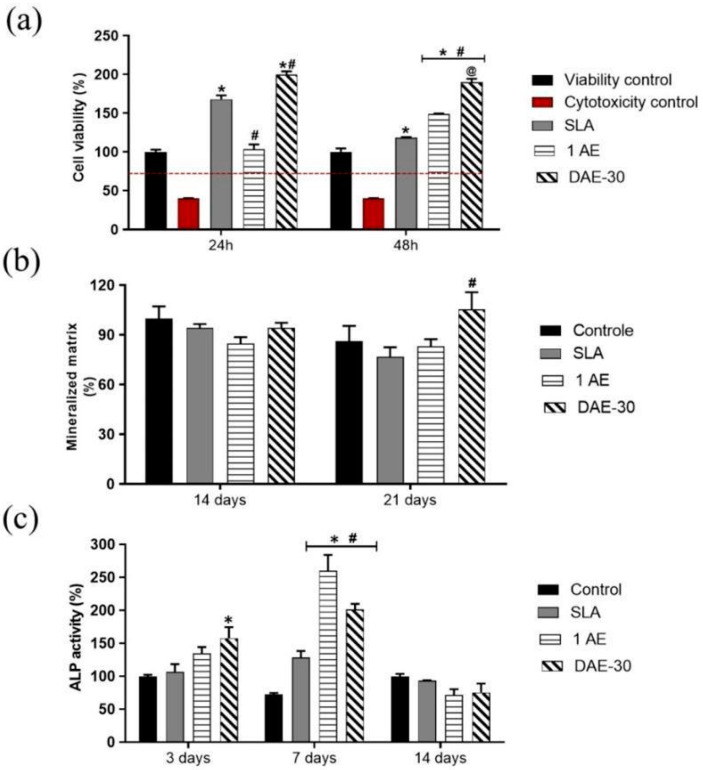
(**a**) MTT assay showing the viability of MC3T3-E1 cells after 24 and 48 h. (**b**) Extracellular matrix mineralization after 10, 14, and 21 days of culture. (**c**) ALP activity measured after 3, 7, and 14 days. * *p* < 0.05 vs. viability control; # *p* < 0.05 vs. SLA; @ *p* < 0.05 vs. 1AE. ALP, alkaline phosphatase.

The results of cell-viability tests are shown in Figure 11a. All sample groups showed >70% cell viability, indicating that the samples were viable and non-toxic to pre-osteoblastic MC3T3-E1 cells. Notably, all the studied surfaces showed cell viabilities higher than 70%. The 48 h viability values for the non-toxic SLA group (118.16 ± 1.62) were lower than those of all the other groups (1AE, 148.85 ± 1.21; DAE-30, 190.15 ± 6.08)

Figure 11b presents the extent of extracellular matrix mineralization observed on the TiO_2_ surfaces after 10, 14, or 21 days of cell culture. The quantity of mineralized matrix for all groups was not statistically different from that of the control after 14 days. After 21 days, the DAE-30 (105.35 ± 17.98) group showed a significant increase in the mineralized matrix amount compared with those of the other groups, demonstrating that this surface tended to induce an improved osteogenic response compared with that induced by the SLA (76.75 ± 10.04) or 1AE (83.19 ± 7.42) surfaces.

Figure 11c presents the results for the ALP activity assay. The SLA surface exhibited higher roughness and lower ALP production. Surfaces with lower roughness (1AE and DAE-30) showed significantly higher ALP levels (260.31 ± 33.67 and 201.58 ± 14.54, respectively) than the SLA (128.48 ± 13.91) group, indicating enhanced cell differentiation.

## 4. Discussion

We demonstrated that DAE groups may be a good option to replace the SLA method for the surface modification of commercial dental implants. They have the potential to replace the commercially used but undesirable surface-modification method SLA.

As previously reported [25,26,27], the SLA surface is characterized by the presence of roughness at the micrometric level, but not at the nanometric level. This was demonstrated via SEM (Figure 4), and no nanometric features were observed on the SLA surfaces. However, the surfaces of the samples modified by acid etching (Figure 4b–e) displayed both micrometric and nanometric pores on their surfaces. This was previously observed by Hung et al. (2017) [27], who also affirmed that such pores serve as a support to the tentacles of osteoblast cells penetrating deeper into the surface, thereby increasing stability after implantation.

As indicated by the Wenzel equation [28], higher roughness values tend to generate surfaces with lower contact angles. However, wettability is dependent on various surface qualities [29]; a comparative study of SLA and nanotubular surfaces [30] showed that despite the higher roughness of SLA surfaces than the surfaces featuring TiO_2_ nanotubes, SLA surfaces exhibited higher contact angles. This corroborates the hypothesis that roughness is not the only determining factor for TiO_2_ surface hydrophilicity, as evidenced in the present study.

During the immersion test, complete deposition coverage was observed for all groups as indicated in previous studies after 28 days [22]. The EDS results were as expected owing to the presence of TiO_2_ layer [25], suggesting that no significant chemical differences were detected among the surface treatments. Furthermore, the spots observed in the DAE-30 group may indicate that this surface has a high tendency for mineral deposition, including HA [31], which was further verified in this study through the mineralized-matrix test. The HA peak obtained in our XRD experiments has also been observed by other researchers [32,33,34] at approximately 31°, which reinforced the presence of HA in samples subjected to the SBF immersion test. The large width of the HA peaks in the diffractograms indicated that low-crystalline HA, favoring osseointegration, was deposited on the surface owing to increased surface free energy and stimulation of osteoblast differentiation-related biochemical marker expression [35,36]. Such low-crystalline HA formation is commonly observed in bioactive materials, such as cp-Ti, A-W glass ceramics, and NaOH- and heat-treated Ti metals [37].

Furthermore, the needle-like structures observed in the electron micrographs (Figure 10a–c, left side) resemble those observed in other studies [32,33,34] related to HA layer growth on A-W bioactive ceramic substrates. The presence of titanium and oxygen on the surface can be attributed to the spontaneous formation of a titanium-oxide layer on the surface. Considering the atomic quantities of calcium and phosphorus in the sample, the calcium-to-phosphorus ratio of 1.67 expected for HA (Ca_10_(PO_4_)_6_(OH)_2_) [38,39,40] was close to that observed by analyzing the semi-quantitative EDS results. Our results also align with those from studies on HA coatings on Ti-alloy surfaces after SBF immersion [35,36,38].

As widely demonstrated in the scientific literature, physicochemical and morphological changes on the surface of bone implants can positively impact cell adhesion and, consequently, cellular viability [30,31,41]. To investigate this effect, an MTT-based cell viability assay was performed. It revealed favorable results, indicating high cellular viability in all analyzed groups (Figure 11a). Notably, the viability was particularly high on 1AE and DAE-30 surfaces. Given that the SLA surface has non-toxic characteristics [31], the acid-etched surfaces were both non-toxic and more viable than the SLA surface, although the presence of water and saliva in the oral cavity lowers the risk of citotoxicity [42]. Thus, reduced roughness did not compromise cell growth [43]. These results can be attributed to the fact that, despite being smoother than SLA surface, the surfaces treated with acid etching exhibited nanometric pores that promote cell adhesion, proliferation, and viability [44,45].

The ALP activity results also corroborate the data from literature, as the SLA surface showed higher roughness and consequently lower ALP production, and surfaces with lower roughness values showed significant ALP levels [46]. Additionally, the observed parameters, combined with a lower contact angle, contributed to the differentiation of pre-osteoblastic cells. This effect was particularly notable in the DAE-30 group, which showed a significant and early increase in cellular differentiation by day 3 of evaluation; this effect was observed in both the DAE-30 and 1AE groups (Figure 11c) till day 7. These findings indicate that the 1AE and DAE-30 surfaces possess characteristics that may promote osteogenic differentiation.

Corroborating these results, more amount of mineralized matrix was deposited on the DAE-30 surface (Figure 11b) than on the SLA or 1AE surfaces. The quantification of this matrix was performed using Alizarin red staining, which revealed a significant increase in calcium salt deposition, indicating higher extracellular matrix mineralization.

These findings reinforce the potential of the 1AE and DAE-30 surfaces in promoting osteogenic processes and their relevance for clinical applications in implantology.

Surface treatment is one of the most explored possibilities for improving dental implants, as we can accelerate osseointegration, improve primary stability, and improve the manufacturing process, reducing costs and waste production. The results of this study go in the same direction, corroborating other studies in the area and indicating the need for future clinical studies that prove the results of this in vitro study.

## 5. Limitations in Research and Future Perspectives

This study has potential limitations. Increased observing periods may be relevant for the biological assays to better understand long-term biological responses. Additionally, in vivo and clinical tests must be performed to confidently demonstrate the superior performance of acid etching dental implant modification over SLA.

## 6. Conclusions

The results of this study indicate that acid etching presents a viable alternative to the SLA method for surface modification of Ti dental implants. This conclusion is supported by the observed early hydroxyapatite deposition on acid-etched surfaces, especially for the DAE-30 group (after 3 days), after soaked in SBF, in comparison to SLA-treated surfaces. Additionally, acid-etched surfaces demonstrated a significantly higher level of cell viability and differentiation after a seven-day period, further suggesting the superior biological performance of acid etching. These findings underscore the potential of acid etching to offer improved surface bioactivity and biocompatibility, positioning it as a promising technique for biomedical applications.

## Figures and Tables

**Table 1:** Sample groups and respective surface treatments.

Group	Surface Treatment
SLA	Sandblasted + NaOH + HNO_3_ and HF
1AE	H_2_SO_4_ + HCl at 60 °C for 60 min
DAE-10	H_2_SO_4_ + HCl at 60 °C for 60 min + HNO_3_ and HF at 25 °C (10 s)
DAE-20	H_2_SO_4_ + HCl at 60 °C for 60 min + HNO_3_ and HF a 25 °C (20 s)
DAE-30	H_2_SO_4_ + HCl at 60 °C for 60 min + HNO_3_ and HF a 25 °C (30 s)

SLA, sandblasted and acid-etched; 1AE, single-acid-etched; DAE, double-acid-etched.

## Data Availability

The original contributions presented in this study are included in the article. Further inquiries can be directed to the corresponding authors.
